# Biomimetic Liquid Metal–Elastomer Composited Foam with Adjustable Thermal Conductivity for Heat Control

**DOI:** 10.3390/molecules28041688

**Published:** 2023-02-10

**Authors:** Hongyao Tang, Xiaozhou Lü, Xiangyu Meng, Hai Wang, Guanghui Bai, Weimin Bao

**Affiliations:** 1 School of Aerospace Science and Technology, Xidian University, Xi’an 710071, China; 2 Science and Technology on Space Physics Laboratory, Beijing 100076, China

**Keywords:** adjustable thermal conductivity material, thermal management, soft composite, liquid metal

## Abstract

The application of traditional materials with constant thermal conductivity in time-varying thermal environments poses great challenges due to their inability of adjusting thermal conductivity according to different requirements, for which reason materials with adjustable thermal conductivity have attracted much attention. However, certain limitations induced by those materials’ low softness or harsh adjustment conditions restrict them from being applied in heat dissipation and heat transfer scenarios. In this study, we report a biomimetic liquid metal–elastomer composited foam with adjustable thermal conductivity (B-LM-ECF). Inspired by the rationale of homeothermic animals regulating the thermal conductivity of their subcutaneous tissue, the prepared material adjusts its thermal conductivity via adjusting the volume proportion of liquid metal within it. The thermal conductivity of B-LM-ECF can be adjusted within the range of 0.11–8.4 W·m^−1^K^−1^. The adjustment factor *η* of B-LM-ECF is 76, which is defined as the ratio of the highest to the lowest thermal conductivity of the material. The material enabling reversible switching for itself from thermal insulation to heat dissipation. The prepared material exhibits 45 KPa of Young’s modulus with the maximum fracture tensile rate of 600%, facilitating better covering for thermal management objects. We selected a power lithium battery and a smartphone as specific thermal management objects to demonstrate its practical application in thermal management experiment.

## 1. Introduction

Thermal conductivity is one of a material’s most critical thermal parameters, which reveals a material’s capability of transferring heat [1,2,3]. On the one hand, low thermal conductivity materials have been widely used in thermal insulation and preservation, such as polymer foam [4,5,6,7,8], fiber [9,10], aerogel [11,12,13,14,15], and polymer film [16,17,18,19,20]. On the other hand, high thermal conductivity materials have greatly valued in heat dissipation and transfer, such as metal [21,22,23,24,25], graphene [26,27,28,29,30,31,32], and diamond [33,34,35,36,37,38]. However, when the ambient temperature or energy consumption of heat sources varies with time, materials with constant thermal conductivity cannot satisfy the requirement of in-time switching between heat dissipation and heat preservation [39]. Furthermore, the drastic change in ambient temperature severely impacts certain devices in their service life and performance, particularly in precision electronics and circuits, and in automobile power lithium batteries.

Therefore, the materials characterizing with adjustable thermal conductivity have attracted much attention in studying thermal conductivity materials [39,40,41,42,43,44,45,46,47,48,49]. Cahill et al. reported a lithium cobalt oxide with adjustable thermal conductivity, which is capable of dynamically adjusting its thermal conductivity by controlling the concentration of lithium ions in the range of 3.7–5.4 W·m^−1^K^−1^ with the adjustment factor $\left(\eta =\frac{k_{max}}{k_{min}}\right)$ of 1.46 [39]. Yildiz et al. reported a lanthanum-strontium cobalt oxide, which can regulate the ion insertion rate in molecular structure by electrochemical interaction, thereby realizing adjustable conductivity for the material in the range of 0.44–4.4 W·m^−1^K^−1^ with η=10 [40]. Zhang et al. reported a hexagonal nickel sulfide with variable thermal conductivity. The material’s thermal conductivity varies dramatically in a specific temperature range, so it can be adjusted via temperature. The material can be adjusted in the field of 4.0–12.0 W·m^−1^K^−1^ with an adjustment factor of 3 [41]. Chen et al. reported a graphite/hexadecane suspension with variable thermal conductivity. During the phase transition, the thermal resistance of the interface between the hexadecane and the graphite filler changes due to the change of internal stress. As a result, the thermal conductivity of the material changes dramatically during the phase transition, from 0.3 to 0.9 [49]. Zheng et al. reported a carbon nanotube/hexadecane composite. As the material transitions from liquid to solid, the carbon nanotubes in the material are pushed to the lattice boundary due to internal stresses. The distribution of carbon nanotube packing will affect the thermal conductivity of the material. Thus, the thermal conductivity of the material can be changed from 0.15 to 0.37. The material has an adjustment factor of 2.46 [47].

The above excellent works have contributed to developing adjustable thermal conductivity materials. However, the above adjustable thermal conductivity materials are all rigid and hard materials, which are difficult to fit the heat dissipated objects. There are still significant challenges in applying them to heat dissipation and heat transfer. Wang et al. proposed a liquid metal composite open-hole sponge that realizes adjustable thermal conductivity via compression deformation in the range of 0.51–4.25 W·m^−1^K^−1^ with an adjustment factor of 8.3 [42]. By changing the contact state between the graphene layers through the change in air humidity, Zhang et al. prepared a graphene-silk fabric with adjustable thermal conductivity. The material’s thermal conductivity variation range is 5.4 to 75 W·m^−1^K^−1^ with the adjustment factor of 14.1 [43]. We have previously reported a flexible thermal management material, but the thermal conductivity of the material is not adjustable [50]. The above studies paved a creative way to adjusting the thermal conductivity of flexible materials. Although the above researches are creative ways to adjust the thermal conductivity of flexible materials, they relied on harsh adjustment conditions (maintaining the compression state or specific ambient humidity), which makes their application in practical applications still a big challenge.

**Figure 1:**
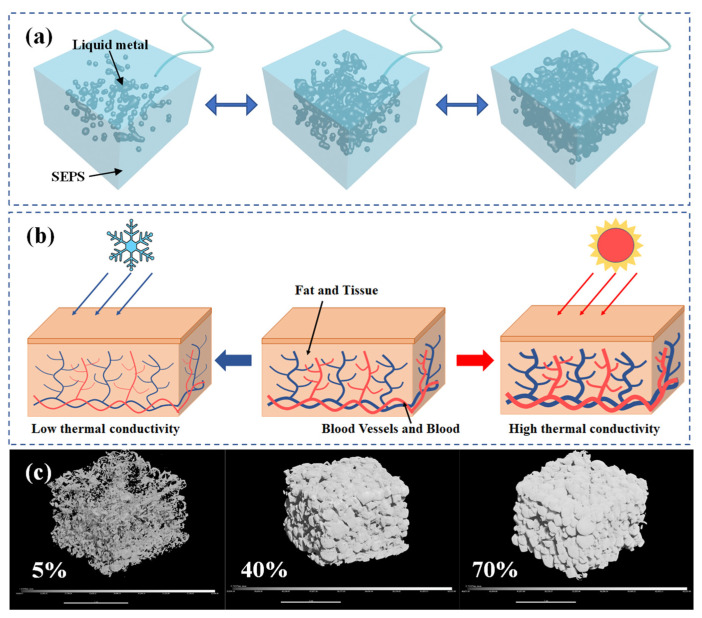
(**a**) B-LM-ECF regulating thermal conductivity by changing liquid metal content, (**b**) Subcutaneous tissue regulating thermal conductivity by changing blood content; (**c**) Micro-CT diagram of B-LM-ECF with 5%, 40%, and 70% liquid metal volume percentage.

Furthermore, we note that endotherms are capable of regulating the thermal conductivity of their own tissues. Homothermic animals, such as human beings, can change the thermal conductivity of their subcutaneous tissue by adjusting the amount of fat, connective tissue, and blood in the subcutaneous tissue to protect themselves against environmental changes. When being in high-temperature environment, the capillaries of human body will expand, causing the blood with high thermal conductivity to occupy more volume, which increases the thermal conductivity of biological tissues. When being in low-temperature environment, the capillaries will shrink, which results in connective tissue with low thermal conductivity, and makes fat to occupy more volume, thereby reducing the thermal conductivity of biological tissues. The regulation mechanism is demonstrated in Figure 1b. This principle has inspired us to make similar biomimetic materials. In this study, we propose a biomimetic liquid metal–elastomer composited foam (B-LM-ECF) that is characterized with adjustable thermal conductivity. Inspired by the above mechanism and other excellent bioinspired responsive soft materials [51,52,53,54,55,56], high thermal conductivity (=26.6 W·m^−1^K^−1^) liquid metal (gallium indium alloy) was therefore selected to fabricate the B-LM-ECF, to which soft and porous material SEBS with low thermal conductivity was selected as the substrate.

In order to further explore the rationale of thermal conductivity regulation of composite materials, we adopted finite element simulation method to verify the theoretical correctness of thermal conductivity regulation. We selected power lithium batterie and smartphone as specific thermal management objects to conduct experiments, demonstrating the application of B-LM-ECF in practical thermal management. The experimental results reveal that the B-LM-ECF is capable of satisfying the expected transformation of heat dissipation and thermal insulation of power lithium batteries under different working states and environments. When the material is applied to smartphones, it can dissipate heat for high-temperature phones in high thermal conductivity state, and can adaptively switch itself to low thermal conductivity state for heat dissipation under low-temperature environment. The above experiments suggest that the proposed B-LM-ECF exhibits excellent thermal management capability in diverse typical thermal management situations, and has great application prospects in niche markets in the field of heat control.

## 2. Results and Discussion

### 2.1. Materials

The materials we used in this study are presented as follows: EGaln (Ga:In = 75:25) was purchased from Dingtai Metal Material Co., Houjie, Dongguan City, China. Styrene-ethylene-propylene-styrene (SEPS) Thermoplastic elastomer material was purchased from Shenzhen Jiushuo Plastic Technology Co., Ltd, Shenzhen, China. Deionized water was purchased from Xi’an Haimeng Material Co., Ltd, Xi’an, China. Salt particles (particle size: 300–500 μm) were purchased from China National Salt Industry Corporation, Xi’an, China. Injection pipes were purchased from Xi’an Haimeng Consumables Co., Ltd, Xi’an, China.

### 2.2. Fabrication of the B-LM-ECF

The preparation process of B-LM-ECF can be subdivided into two steps: the preparation of SEPS porous matrix, and the injection and control of liquid metal. First, we prepared the SEPS porous matrix using salt template method. Specifically, we sprayed the salt particles with water and pressed them into a salt block by using a mold, then used a hot air gun to dry the excess water of the salt block to make the salt particles condense and conjugate with each other, forming a salt template. While preparing the salt template, we put the SEPS particles into a beaker and heated them to 150 degrees Celsius to make them fluid. It should be noted that the fluidity of SEPS increases with temperature. SEPS become fluid at 120 degrees Celsius. However, at 120 degrees Celsius, the SEPS fluid is too viscous to flow into the gaps in the salt template. Thus, we had to heat the SEPS to 150 degrees Celsius to ensure that the SEPS fluid could flow into the salt template gap. Then, we put the salt block into the molten SEPS fluid and kept it heated. The salt template bubbled as it heats, due to the SEPS flu-id constantly filled the gaps in the salt template. Once the salt template was no longer bubbling, we cooled the SEPS. After the SEPS was solidified into an elastomer, we used deionizing water to remove the salt blocks, thereby obtaining SEPS porous substrate. Then, we connected the injection hose with the SEPS porous substrate and injected liquid metal into the substrate. The outstanding work of Whitesides et al. has given us inspiration [57]. When the liquid metal filled the pores of the SEPS porous substrate material, the preparation of B-LM-ECF is finished. The detailed preparation process can be referred to our previous work [50]. The thermal conductivity of the B-LM-ECF was regulated by an injection regulator device connected to the B-LM-ECF through an injection hose to control the volume content of liquid metal within it. The external injection device can be flexibly changed according to different engineering applications, such as peristaltic pumps, medical syringes, and diaphragm pumps. In this study, we selected the peristaltic pump as the injection adjustment device of B-LM-ECF.

### 2.3. Characterizations and Measurements

We used a micro-CT device (MODEL: ZEISS Xradia 610 Versa) to characterize the as-prepared B-LM-ECF at different liquid metal volume ratios. Light microscopy (MODEL: Phenix XTL-165-VT) was used to observe the cross-section of B-LM-ECF with high and low liquid metal content. Since the optical cross-section observation experiment is destructive, we selected two pieces of B-LM-ECF. The Young’s modulus and the maximum fracture lift rate of B-LM-ECF were tested by material universal tensile testing machine (MODEL: ZHIQU ZQ-980). The experimental samples with 30 mm length, 20 mm width, and 9 mm thickness were loaded and fixed onto the testing machine. The thermal conductivity of B-LM-ECF was characterized by a thermal conductivity measurement device. The device was composed of 25 μm diameter platinum wire, copper electrode, polyimide insulating tape, and digital source table SUM2450, according to the guidance of our previous papers. The thermal conductivity of B-LM-ECF at different liquid metal content is averaged by repeating three measurements. For details, see Table S1.

### 2.4. Mechanical Properties and Micromorphology of B-LM-ECF

With respect to the B-LM-ECF with different liquid metal volume ratios of 5%, 40%, and 70%, the distribution of liquid metal under micro-CT is shown in Figure 1c. It can be observed that under the state of 5%, the liquid metals in B-LM-ECF exhibited isolated structures of discretized points without mutual connectivity. Under 40% state, the liquid metals in B-LM-ECF exhibited a network-like structure with partial connection, whereas certain liquid metals still remained separate without being connected. Under 70% state, the liquid metals in B-LM-ECF displayed a fully connected network structure. The above three states demonstrates the process of liquid metals in B-LM-ECF from separate to partially connected, then to fully connected, reflecting the rationale of the adjustable thermal conductivity of B-LM-ECF.

**Figure 2:**
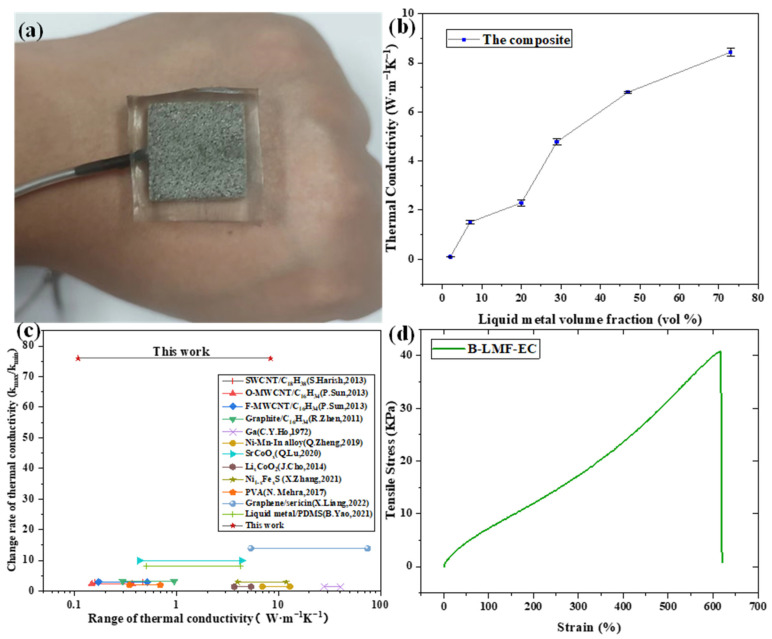
(**a**) Physical image of B-LM-ECF. (**b**) Variation curve of the adjustable thermal conductivity of B-LM-ECF with different liquid metal volume fraction. (**c**) Comparison of the performance of existing materials with adjustable thermal conductivity [39,40,41,42,43,44,45,46,47,48,49]. (**d**) Maximum tensile fracture curve of the B-LM-ECF.

The sectional optical photos of B-LM-ECF with a high liquid metal volume ratio (70%), and a low ratio (2%) are shown in Figure 2c,d, respectively. It can be observed that under the circumstance of low liquid metal volume ratio of the as-prepared B-LM-ECF material, those liquid metals are scattered and separated with each other, for which reason continuous thermal conduction pathway cannot be formed. In contrast, in the material with high liquid metal volume ratio, those metals are interconnected and closely attached to the SEPS substrate, thereby forming a continuous thermal conduction pathway.

**Figure 3:**
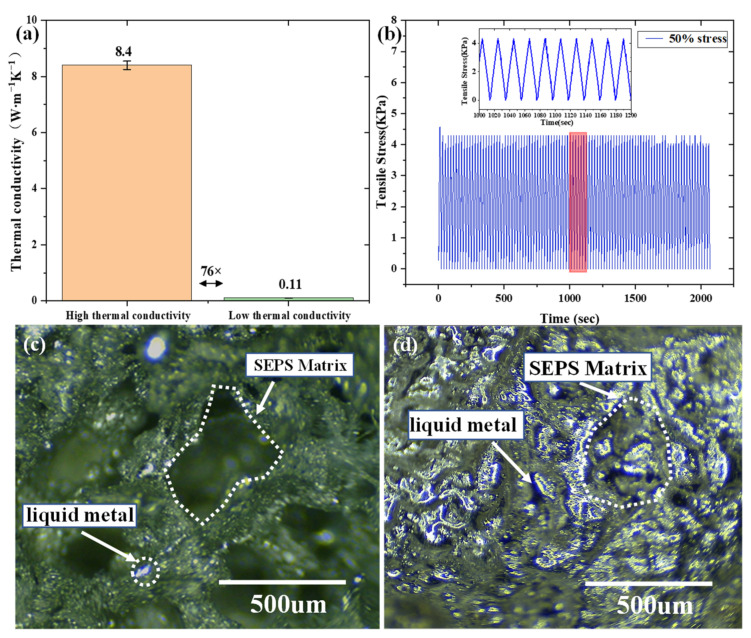
(**a**) Comparison of the thermal conductivity of B-LM-ECF at the high and low thermal conductivity. (**b**) Graph of 100 cyclic stretching experiments of B-LM-ECF. Optical micrograph of the section of B-LM-ECF with (**c**) low thermal conductivity and (**d**) high thermal conductivity.

To verify the softness and stretchability of the as-prepared material, its Young’s modulus was measured, to which the test results are shown in Figure 2d. We observe that the material’s maximum tensile rate is 600% Without loss of generality, the B-LM-ECF with 70% liquid metal volume ratio was selected for cyclic tensile experiment, and the experimental results are shown in Figure 3b. The mechanical properties of the B-LM-ECF remains unchanged in 100 cyclic tensile tests.

### 2.5. Mechanism of the Thermal Regulating of B-LM-ECF

In order to better explain the mechanism of material thermal conductivity adjustment, we decided to use numerical calculation methods to calculate the thermal conductivity of materials under different liquid metal content. However, currently, there are many numerical calculation methods. We must choose a suitable method to calculate the thermal conductivity of B-LM-ECF.

Scientists have been using numerical methods to estimate the thermal conductivity of composite materials since the 20th century. These numerical methods fall into two broad categories: classical models and computer-based finite element models. The most commonly used models of classical thermal conductivity prediction are the Maxwell model, the Rayleigh model, the Hasselman–Johnson model, and the Bruggeman model. The Maxwell model assumes that spherical particles with high thermal conductivity are uniformly dispersed in the continuum, the particles do not contact each other, and there is no thermal resistance between the particles and the continuum. Since the contact and interfacial thermal resistance of particles are not considered, the Maxwell model will have a large error in predicting the thermal conductivity of materials with higher filling proportions. The Rayleigh model is a two-dimensional structural material model, which has good prediction results for two-dimensional structural materials but has large prediction errors for three-dimensional structural materials. The Hasselman–Johnson model is an improved model of the Maxwell model. The Hasselman-Johnson takes into account the effect of interfacial thermal resistance on the thermal conductivity of composites for the first time, which improves the accuracy of the model prediction. However, the Hasselman–Johnson model does not take into account the effect of fillers’ contact with each other. Therefore, there is still a large error in the prediction of the thermal conductivity of composites with high filling proportions. The Bruggeman model creatively uses differential effective medium theory, which makes its prediction accuracy improve. However, the model still difficult to predict the thermal conductivity of composites with continuous heat transfer networks. According to the above analysis, we find that the classical model is not suitable for predicting the thermal conductivity of B-LM-ECF. Thus, we turned our attention to finite element analysis methods. In recent years, with the rapid development of computer technology, the computing power available to scientists has increased exponentially. Therefore, using finite element analysis software to predict the thermal conductivity of composite materials has become a new trend in research.

**Figure 4:**
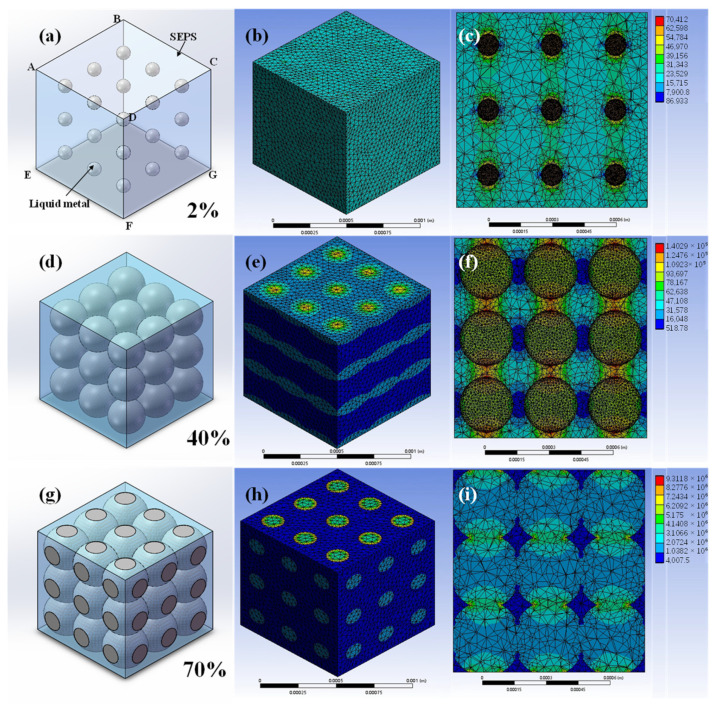
(**a**,**d**,**g**) Geometric models of composites with different liquid metal proportions(2%, 40%, and 70%). (**b**,**e**,**h**) finite element analysis on heat flux diagram of the B-LM-ECF with different liquid metal proportions. (**c**,**f**,**i**) cross-section heat flux diagram for finite element analysis on the B-LM-ECF with different liquid metal proportions.

Therefore, we selected finite element simulation method to simulate the B-LM-ECF with different liquid metal volume content, to which, the results obtained under different liquid metal contents are shown in Figure 4. As shown in Figure 4a, we set the upper surface (ABCD) of the model as a constant temperature surface of 100 °C, the lower surface (EFGH) as a constant temperature surface of 20 °C, and the rest of the surface as an adiabatic surface, thereby creating a temperature difference of 80 °C. According to the finite element simulation results, the average heat flux in the model is obtained. According to Fourier’s law:

$$q=-k\cdot \left(\frac{\Delta T}{\Delta x}\right)$$

we can derive an equation for calculating the thermal conductivity:

$$k=-\frac{q}{\left(\frac{\Delta T}{\Delta x}\right)}$$

In the above equation, *k* is the thermal conductivity of the composite material, *q* is the heat flux, ΔT is the temperature difference between upper and lower limits, Δx is the distance between upper and lower surfaces. The negative sign indicates the directionality.

The simulated heat conductivity of the B-LM-ECF is calculated by the percentage of different liquid metal volumes, which is shown in Figure S2. It can be observed that the finite element simulation results are in good consistency with the actual thermal conductivity. However, due to the impact of interface thermal resistance on the thermal conductivity of the composite materials, the actual thermal conductivity is lower than the simulated thermal conductivity.

### 2.6. Thermal Properties and Applications of B-LM-ECF

The thermal conductivity of the material is the property that we are most concerned about. Therefore, it is necessary to choose the appropriate thermal conductivity measurement method to measure the thermal conductivity of B-LM-ECF. The thermal conductivity measurement methods of materials are mainly divided into two categories: steady state measurement method and transient measurement method. Steady-state thermal conductivity measurement methods include the guarded hot plate method, heat flow meter method, hot box method, etc. Transient thermal conductivity measurement methods include the hot wire method, hot-disk method, laser flash method, photothermal method, etc. The steady-state measurement method has the advantage of high measurement accuracy. However, the steady-state method is time-consuming and requires expensive equipment and elaborate experimental design. Therefore, the transient measurement method is more consideration for the thermal conductivity measurement of composite materials. The main idea of the transient method is to apply a transient thermal change to the object to be measured, then calculate the thermal conductivity according to the response of the object to be measured. The laser flash heat method is to apply a transient laser heat pulse to the material under test using a specific test machine. Then, the thermal conductivity of the material is calculated according to the temperature change and density of the material. The laser flash heat method has the advantages of simple operation and fast test speed. However, B-LM-ECF contains liquid metal, which risks contaminating the test machine. The transient hot wire method is the most commonly used method to measure transient thermal conductivity. The principle of the method is to use a thin metal wire to heat the material under test. Then, the thermal conductivity of the measured material is calculated by heating power, metal wire length, and material temperature change rate. The method is simple and short in time and enables accurate measurements of materials containing liquid. Therefore, the transient hot-wire method is considered a suitable method to measure the thermal conductivity of composites containing liquid metals. The hot-disk method is an improvement of the transient hot-wire method. The hot wire in the transient hot wire method is replaced by a plane composed of metal wires. This change simplifies the operation required for the hot-disk method. Therefore, the operator is not required to learn professional knowledge related to heat transfer, and the machine can automatically measure thermal conductivity. However, the shape of the thermal conductivity probe in the hot-disk method is fixed and cannot be adjusted according to the shape of the measured material. The test instruments of the hot-disk method are usually commercial finished instruments, which are difficult to change. Therefore, although the method is simpler to test, it is not as flexible and easy to adjust as the transient hot wire.

Based on the above analysis, we decided to use the transient hot wire method to measure the thermal conductivity of materials. Specifically, an insulated platinum wire with 25 μm diameter was placed in the liquid metal foam, then both ends of the foam were connected to the digital source meter SUM2450 with copper electric wires. Subsequently, the platinum wire was energized with a step current from the source meter, during which 50 voltage values within 1 s were recorded. To eliminate errors and noises, we repeated the evaluation three times at an interval of 5 min. The resistance of the platinum wire was then calculated according to the voltage and current values, and the temperature rise of the wire was calculated via using the resistivity temperature coefficient of the platinum. By using the obtained points from the experimental data, the temperature-time logarithmic curve of the platinum wire can be fitted, after which the straight-line interval in the curve was selected to calculate and the slope of the straight line.

The thermal conductivity of the prepared TSH-LMF-EC material can be obtained by using the following thermal conductivity calculation formula of transient hot-wire method:

$$k=\frac{UI}{4\pi L\left(\frac{dT}{d\;lnt}\right)}$$

In the above equation, d*T*/(*d lnt*) denotes the slope of the straight line, *L* denotes the length of the hot wire, and *U* and *I*, respectively, denote the voltage and current values.

We measured the thermal conductivity of the as-prepared B-LM-ECF with 2%, 6%, 20%, 29%, 47%, and 73% liquid metal volume ratios, to which the test results are shown in Figure 2b. It can be observed that the thermal conductivity of B-LM-ECF varies from 0.11 to 8.4 W·m^−1^K^−1^ as the liquid metal volume ratio changes. Under the circumstance of low liquid metal content, the thermal conductivity of B-LM-ECF is even lower than that of pure SEPS block due to the thermal insulation effect of the porous foam structure.

To demonstrate the application of B-LM-ECF in the practical field, we selected power lithium batteries and smartphones as specific heat dissipation objects to conduct heat management experiments.

**Figure 5:**
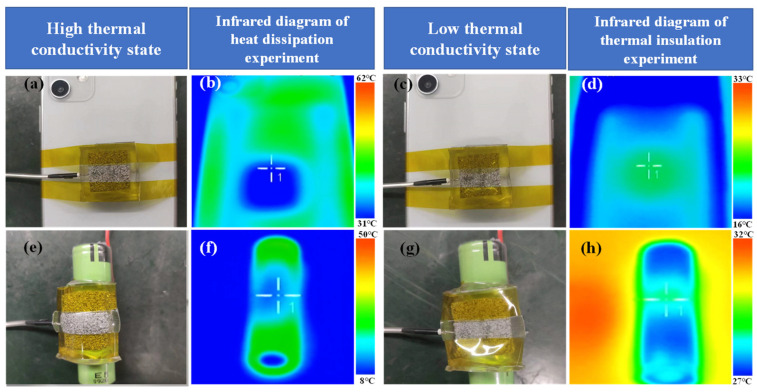
(**a**,**b**) physical and infrared images of high thermal conductivity B-LM-ECF on smartphone for thermal insulation. (**c**,**d**) physical and infrared images of low thermal conductivity B-LM-ECF on smartphone for thermal insulation. (**e**,**f**) physical and infrared images of high thermal conductivity B-LM-ECF on lithium battery for thermal insulation. (**g**,**h**) physical and infrared images of low thermal conductivity B-LM-ECF on lithium battery for thermal insulation.

The power lithium battery shall be kept within appropriate temperature range. Low temperature will cause the decline of ion activity in lithium battery, resulting in low battery power or even failure to start. High temperature may induce spontaneous combustion and/or even implode of the electrolyte in the battery. For concrete cases of using electric vehicles, in the process of charging or driving a vehicle, high thermal conductivity materials are required to dissipate heat for power batteries to take precautions against spontaneous combustion, and/or explosion of vehicles. When the vehicle is parked in low-temperature environment, such as in winter and at night, low thermal conductivity materials help keeping the power battery warm for longer endurance mileage and easy start. This practical requirement demands that thermal management materials must be enabled with adjustable thermal conductivity to cope with different situations and environments. To verify the thermal management capability of B-LM-ECF on powerful lithium battery, we wrapped the material on a cylindrical 18,650 power lithium battery for the experiment, to which the corresponding physical images are shown in Figure 5e,h. During the process of lithium-ion charging, we set B-LM-ECF to the state of high thermal conductivity to dissipate heat for lithium batteries. When the battery is in cold wind environment, we adjusted the B-LM-ECF to its low thermal conductivity state to keep the battery warm. Figure 5f,h are the infrared thermal images illustrating the heat control of lithium battery, from which we observe that the B-LM-ECF can effectively regulate the temperature of the lithium battery. When the battery is under charging state, the temperature of the coated/wrapped battery decreases compared with that of the unwrapped part. When being placed in low-temperature environment, the temperature of the wrapped battery increases compared with that of the uncoated part. We have collated the thermal conductivity of common foams as shown in Supporting Material Table S2 [58,59,60,61]. We can see that the foam materials have low thermal conductivity. This experiment reflects the application prospect of B-LM-ECF for thermal management of vehicle-powered lithium batteries.

## 3. Conclusions

This study reports a liquid metal foam elastomer composite with adjustable thermal conductivity based on biomimetic mechanism. The thermal conductivity of the as-prepared highly soft flexible material can be adjusted, which covers a wide range and has fast response time, exhibiting great application potential in thermal management devices.

## Data Availability

Data available on request due to restrictions, e.g., privacy, or ethical.

## Supplementary Materials

The following are available online at https://www.mdpi.com/article/10.3390/molecules28041688/s1, Figure S1: Finite element simulation diagram of thermal conductivity of B-LMF-EC with different liquid metal volume percentage, Figure S2: Simulation result curves using finite element method on thermal conductivity regulation principle of B-LMF-EC, Figure S3:The temperature rise versus natural logarithmic time curves of Water, Table S1: Specific data of thermal conductivity test of B-LM-ECF, Table S2: Thermal conductivity of regular foams.

## Sample Availability

Samples of the compounds are available from the authors.
