# Supplementary material for: Biomimetic Liquid Metal–Elastomer Composited Foam with Adjustable Thermal Conductivity for Heat Control
Source: Molecules. 2023 Feb 10;28(4):1688. doi: 10.3390/molecules28041688 (PMC9962720; doi:10.3390/molecules28041688)
Supplement: Supplementary file 1 [file molecules-28-01688-s001.zip › molecules-2175772-supplementary.pdf]

Supporting Information

Biomimetic Liquid Metal–Elastomer Composited Foam with Adjustable Thermal Conductivity for Heat Control

Hongyao Tang [1], Xiaozhou Lü [1,*], Xiangyu Meng [1], Hai Wang [1], Guanghui Bai [2] and Weimin Bao [1]

1. School of Aerospace Science and Technology, Xidian University, Xi'an 710071, China; hytang@stu.xidian.edu.cn (H.T.); xymeng@stu.xidian.edu.cn (X.M.); wanghai@mail.xidian.edu.cn (H.W.); baoweimin@cashq.ac.cn (W.B.)
2. Science and Technology on Space Physics Laboratory, Beijing 100076, China; bghbuaa@aliyun.com

* Correspondence: xzlu@xidian.edu.cn

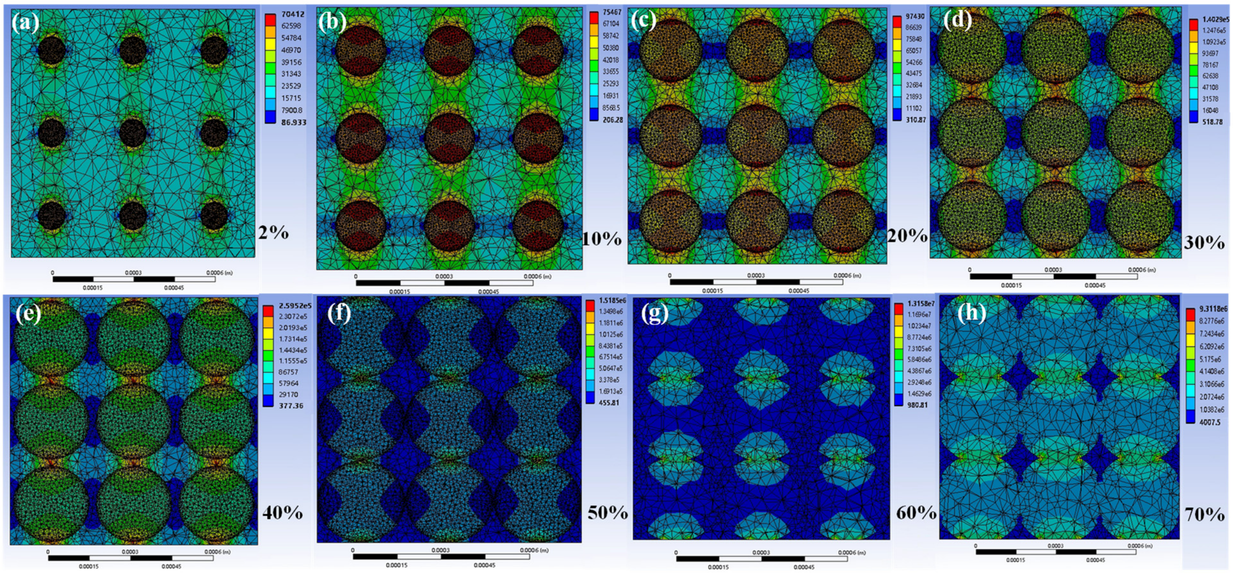

Figure. S1 Cross-section heat flux diagram for finite element analysis on the B-LM-ECF with different liquid metal proportions (2% (**a**), 10% (**b**), 20% (**c**), 30% (**d**), 40% (**e**), 50% (**f**), 60% (**g**),70% (**h**)).

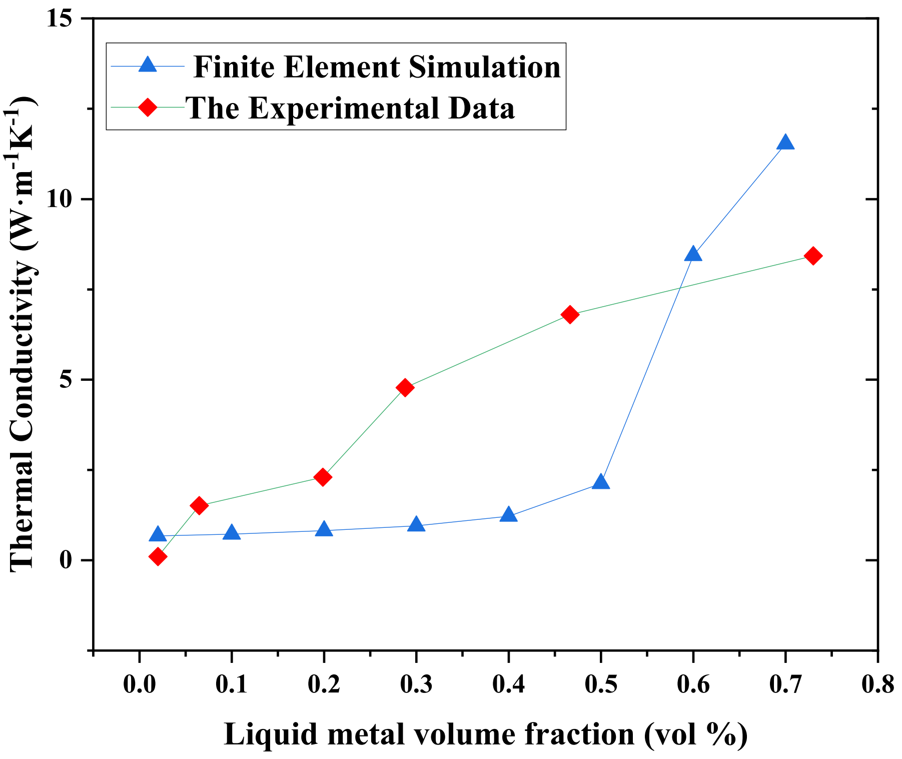

Figure S2. Simulation result curves using finite element method on thermal conductivity regulation principle of B-LMF-EC.

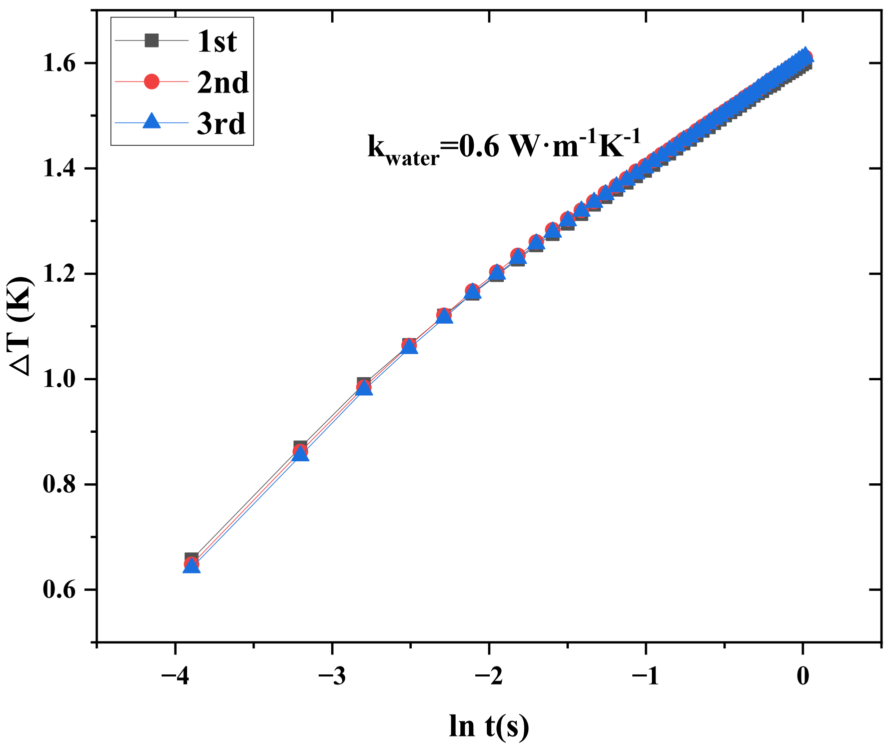

Figure. S3 The temperature rise versus natural logarithmic time curves of Wate

Table S1 Specific data of thermal conductivity test of B-LM-ECF

Table S1 Specific data of thermal conductivity test of B-LM-ECF					
**Liquid metal percentage by volume**	**Thermal conductivity value of the first test**	**Thermal conductivity value of the second test**	**Thermal conductivity value of the third test**	**Average thermal conductivity($W \cdot m^{-1}K^{-1}$)**	**Standard deviation**
2%	0.104	0.107	0.106	0.105	0.001247219
7%	1.62	1.49	1.48	1.53	0.063770422
20%	2.35	2.45	2.15	2.31	0.124721913
29%	4.92	4.62	4.8	4.78	0.12328828
47%	6.76	6.85	6.79	6.80	0.037416574
73%	8.37	8.29	8.64	8.43	0.149740516

Table S2 Thermal conductivity of regular foams

Table S2 Thermal conductivity of regular foams		
**Foam type**	**Thermal conductivity(W·m^{-1}K^{-1})**	**Reference**
PVC foam	0.063	[58]
Polyimide foam	0.029	[59]
Polyurethane foams	0.022	[60]
Polyethylene foams	0.3729	[61]
